# The Impact of Human Pressure and Climate Change on the Habitat Availability and Protection of *Cypripedium* (Orchidaceae) in Northeast China

**DOI:** 10.3390/plants10010084

**Published:** 2021-01-02

**Authors:** Huanchu Liu, Hans Jacquemyn, Xingyuan He, Wei Chen, Yanqing Huang, Shuai Yu, Yupeng Lu, Yue Zhang

**Affiliations:** 1 CAS Key Laboratory of Forest Ecology and Management, Institute of Applied Ecology, Shenyang 110016, China; huanchu.liu@student.kuleuven.be (H.L.); huangyanqing@iae.ac.cn (Y.H.); yushuai@iae.ac.cn (S.Y.); luyp1992@163.com (Y.L.); zhangyue@iae.ac.cn (Y.Z.); 2 College of Resources and Environment, University of Chinese Academy of Sciences, Beijing 101417, China; 3 Department of Biology, Plant Conservation and Population Biology, KU Leuven, B-3001 Leuven, Belgium; hans.jacquemyn@bio.kuleuven.be; 4 Shenyang Arboretum, Chinese Academy of Sciences, Shenyang 110016, China; 5 Institute of Applied Ecology, Chinese Academy of Sciences, 72 Wenhua Road, Shenyang 110016, China

**Keywords:** orchid, geographic distribution, maxent, biodiversity protection

## Abstract

Human pressure on the environment and climate change are two important factors contributing to species decline and overall loss of biodiversity. Orchids may be particularly vulnerable to human-induced losses of habitat and the pervasive impact of global climate change. In this study, we simulated the extent of the suitable habitat of three species of the terrestrial orchid genus *Cypripedium* in northeast China and assessed the impact of human pressure and climate change on the future distribution of these species. *Cypripedium* represents a genus of long-lived terrestrial orchids that contains several species with great ornamental value. Severe habitat destruction and overcollection have led to major population declines in recent decades. Our results showed that at present the most suitable habitats of the three species can be found in Da Xing’an Ling, Xiao Xing’an Ling and in the Changbai Mountains. Human activity was predicted to have the largest impact on species distributions in the Changbai Mountains. In addition, climate change was predicted to lead to a shift in distribution towards higher elevations and to an increased fragmentation of suitable habitats of the three investigated *Cypripedium* species in the study area. These results will be valuable for decision makers to identify areas that are likely to maintain viable *Cypripedium* populations in the future and to develop conservation strategies to protect the remaining populations of these enigmatic orchid species.

## 1. Introduction

Predicting changes in species distributions as a result of human activity and/or climate change is crucial for the conservation and restoration of populations of endangered species [1]. Species distribution models (SDMs) are effective methods for estimating the ecological requirements and potential distribution of species. These models combine individual species’ occurrence records with a set of environmental predictors to estimate the ecological requirements of the species [2,3]. The maximum entropy model (Maxent) has been shown to obtain more accurate prediction results compared with other models when the amount of data used for the prediction is small, making it a valuable tool for the prediction of the potential distribution ranges of endangered species or species of great economic value [4,5,6,7,8].

Human activities in the environment and climate change are considered as two of the most severe threats to the conservation of wild plant and animal species [9,10,11]. Human activities, which includes livestock grazing, urbanization and roads, and has been shown to be important contributors to biodiversity loss [12,13]. Intense human activities can significantly alter the structure of ecosystems, change species distribution patterns and affect ecosystems functions, ultimately leading to a more homogenous biosphere [14,15,16,17]. The impact of human activities on endangered species is predicted to be even more serious, because endangered species are generally more sensitive to changes under human stress than common species [18,19,20]. The Human Footprint (HFP) published in 2016 has become an important tool to study the impact of global human pressure on the environment [21,22,23,24].

Besides human pressure, the distribution of species also depends largely on climatic conditions, suggesting that climate change will affect the future availability of suitable habitat and hence species distributions [25,26,27,28,29]. As a result of climate change, suitable habitats may become increasingly fragmented or disappear altogether, causing further changes in biodiversity [30,31]. Climate change may shrink and/or shift species’ ranges, thereby increasing their vulnerability to external disturbances [32]. It has been predicted that in the next 30–60 years, the climatic conditions will no longer be suitable to support more than 60 species in Chinese nature reserves [33]. Similarly, habitat suitability will decline in more than 130 nature reserves [33]. To survive the changing climatic conditions, species will either have to adapt to the climate change or migrate to other, more suitable habitat areas [34,35,36,37].

Orchids represent one of the largest families of flowering plants in the world, currently containing over 27,000 species [38]. Orchids are widely distributed all over the world, except for extreme deserts and the icy regions that skirt the Arctic and Antarctica [39]. Depending on the scale, their distribution depends on a range of biotic and abiotic factors, including habitat size, light and soil conditions [40], the presence of suitable mycorrhizal fungi [41] and pollinators [42,43,44]. As a result of human interference, many orchids are declining and some of them are highly threatened or have already gone (locally) extinct [45,46]. However, at present there are very few studies that have investigated how the combined impact of land-use change and climate change affects the large-scale distribution of orchids.

In this study, we investigated the impact of human activities on the environment and climate change on the distribution of three *Cypripedium* species in northeast China. In recent decades, overcollection, habitat loss and fragmentation have led to substantial decreases in the distribution and abundance of lady’s slipper orchids in China and elsewhere in the world [47,48,49]. Maxent and ArcGIS were used to assess the importance of multiple environmental variables (including bioclimatic and topographical variables, soil type, vegetation type and human activities) determining the distribution of lady’s slipper orchids in northeast China. In particular, we aimed to (1) assess the relationship between the environmental variables and the distribution of the lady’s slipper orchids; (2) identify the key environmental variables constraining the distribution of *Cypripedium* in northeast China; (3) identify suitable habitats for *Cypripedium*; and (4) assess the effect of human activities and climate change on the distribution of *Cypripedium* in the study area.

## 2. Material and Methods

### 2.1. Study Species

**Figure 1:**
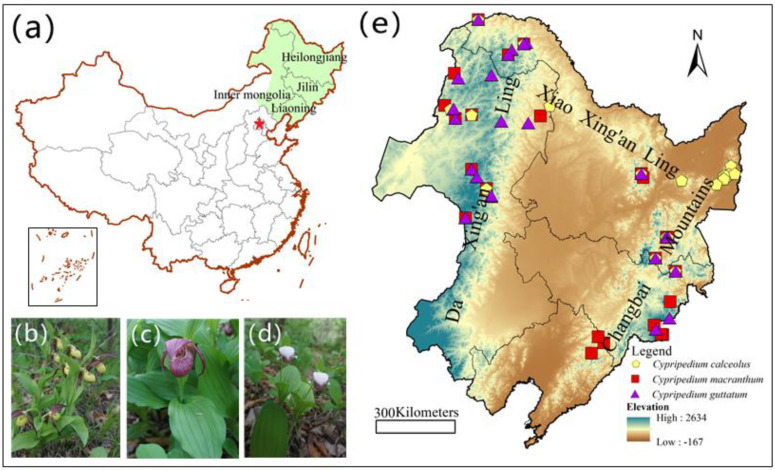
(**a**) Map of China and the study area; (**b**) *Cypripedium calceolus* L.; (**c**) *Cypripedium macranthum* Sw.; (**d**) *Cypripedium guttatum* Sw.; (**e**) geographic locations of *Cypripedium* in northeast China.

*Cypripedium* is a genus of long-lived terrestrial orchids (Orchidaceae) that have distinctive flowers looking like a lady’s slipper. There are about 50 species in this genus, of which 32 species grow in China [50]. They are terrestrial orchids that usually flower between May and July [50]. Most species of *Cypripedium* are distributed in the temperate region of the northern hemisphere [51]. *Cypripedium* species often require very specific climatic conditions for flowering, seed germination and off-spring production [52,53]. Northern China and northeast China are considered as the secondary centers of the distribution of *Cypripedium* in the World [54]. They mainly grow in the broad-leaved or mixed coniferous and broad-leaved forests of northeast China [55,56]. Three species of *Cypripedium* (*C. calceolus* L. (Figure 1b), *C. macranthum* Sw. (Figure 1c) and *C. guttatum* Sw. (Figure 1d)) occur in northeast China and were investigated in this study.

### 2.2. Study Area

The study area is located between 110 and 130° E and between 38 and 50° N in northeast China and occupies approximately 1.2 million square kilometers. This area includes the Liaoning province, Jilin province, Heilongjiang province and the eastern part of the Inner Mongolia autonomic region (Figure 1a). The study area does not completely coincide with the administrative boundary. The climate in northeast China varies between humid in the east and semi-humid monsoon in the west that is affected by the Eastern Asia Monsoon [57]. The mean annual precipitation ranges from 174 to 1124 mm year^−1^ and mean annual temperature ranges from −8.8 to 11.3 °C. The vegetation shows distinct changes with increasing longitude. Da Xing’an Ling and Xiao Xing’an Ling contain mainly cold temperate coniferous forests, the Changbai Mountains harbor mainly temperate broad-leaved deciduous forests, while the Northeastern Plain consists mainly of temperate grasslands [58].

### 2.3. Environmental Niche Modeling

Detailed occurrence records were obtained from field investigations conducted between May 2017 and June 2018 and herbarium records. First, we checked specimens of *Cypripedium* in the Herbarium of the Institute of Applied Ecology, Chinese Academy of Sciences, and used these records as a baseline to plan the survey routes. Surveys were conducted in four northeastern provinces (Heilongjiang, Jilin, Liaoning and the Eastern part of Inner Mongolia) and three mountain ranges (Da Xing’an Ling, Xiao Xing’an Ling and the Changbai Mountains).

The remaining data were obtained from the Global Biodiversity Information Facility (https://www.gbif.org/), the Chinese Virtual Herbarium (http://www.cvh.ac.cn/) and the data sharing platform for forest plant germplasm and habitat survey in northeast China (http://cnes.iae.ac.cn:8888/default.aspx). Occurrence records were removed when they were in the same grid cell or occurred outside the study area. Spatial filtering was used to reduce spatial clumping by keeping one valid occurrence record within grid cells of five kilometers. Finally, a total of 68 validated record points was used in Maxent (Figure 1e), including 19 occurrence records for *C*. *calceolus*, 26 for *C. macranthum* and 23 for *C. guttatum*.

Environmental data were obtained from various databases, including bioclimatic variables from the WorldClim-Global Climate Data (http://www.worldclim.org), the topographic data from Geospatial Data Cloud (http://www.gscloud.cn/), the vegetation data from Resource and Environment Data Cloud Platform (http://www.resdc.cn/DataList.aspx) (vegetation data) and soil data from the Harmonized World Soil Database (http://webarchive.iiasa.ac.at) (soil data). The Human Footprint (HFP) data were downloaded from https://datadryad.org/resource/doi:10.5061/dryad.052q5.2 [59]. The World Database on Protected Areas (WDPA) data (Figure S1a) were provided by the China Council for International Cooperation on Environment and Development. Future bioclimatic variables were provided by IPCC5 (the Intergovernmental Panel on Climate Change) [60]. The future bioclimatic data available were the IPPC5 climate projections from the global climate models (GCMs) for four representative concentration pathways (RCPs). Here, we chose two climate scenarios (rcp4.5 and rcp8.5) in 2070 with the three models of BCC-CSM1-1 (Beijing Climate Center Climate System Model), CCSM4 (The Community Climate System Model) and HadGEM2-AO (Hadley Global Environment Model). Rcp4.5 is a scenario that stabilizes radiative forcing at 4.5 W m^−2^ in the year 2100 without ever exceeding that value by employment of a range of technologies and strategies for reducing greenhouse gas emissions [61]. The rcp8.5 scenario corresponds to a nominal anthropogenic forcing of 8.5 W m^−2^ by 2100, with emissions of CO_2_ following an exponential growth trajectory throughout the 21st century and is generally taken as the basis for worst-case climate change scenarios.

#### 2.3.1. Data Preparation and Selection

The World Climate Database provides 19 bioclimatic variables (Table 1). In addition, we calculated several additional variables, including Kira’s warmth index (WI) [62], Holdridge’s annual biotemperature (ABT) [63], Kira’s coldness index (CI) [62], seasonality of precipitation (PSD) and |WI-CI|.

- Kira’s warmth index (WI) [62],
  WI=∑(T−5) (for months in which T > 5 °C in units of degree month);
- Holdridge’s annual biotemperature (ABT) [63],
  $$\mathrm{ABT}=\frac{\sum T}{12}\;(\mathrm{for}\;\mathrm{months}\;\mathrm{in}\;\mathrm{which}\;T\;>\;0\;{}^{\circ}C\;\mathrm{in}\;\mathrm{units}\;\mathrm{of}\;\mathrm{degree}\;\mathrm{month});$$
- Kira’s coldness index (CI) [62],
  CI=∑(T−5) (for months in which T > 0 °C in units of degree month);
- Seasonality of precipitation (PSD),
  $$\mathrm{PSD}\;=\;\frac{2\sqrt{\frac{{\left(\mathrm{bio}13-\mathrm{bio}14\right)}^{2}}{2}}}{\mathrm{bio}13+\mathrm{bio}14}\;(\mathrm{for}\;\mathrm{bio}13\;=\;\mathrm{precipitation}\;\mathrm{of}\;\mathrm{wettest}\;\mathrm{month},\;\mathrm{bio}14\;=\;\mathrm{precipitation}\;\mathrm{of}\;\mathrm{driest}\;\mathrm{month});$$
- |WI-CI|,
  |WI-CI| = |warmth index − coldness index|.

All these data were calculated using ArcGIS 10.3.

We used Maxent 3.4.1 [64] to construct the environmental niche of each species based on all occurrence records. Maxent sample cells implicitly assume that the actual area of each cell is equal, so the grids should be projected to an equal area projection [65]. All environmental variables (Table 1) were processed as Krasovsky 1940 Albers projection with a resolution of 1 km in ArcGIS. In order to improve the accuracy of the model and to avoid over-fitting, we removed those climatic variables that were highly correlated (Figure S2) and showed low relative contributions in Maxent. Elevation, slope, top soil organic carbon content and top soil pH were selected as the topographic and soil characteristics based on results of the field survey (Table S1 and Figure S3). Finally, a total of nine environmental factors (elevation, slope, isothermality, temperature seasonality, maximum temperature of warmest month, precipitation of driest quarter, seasonality of precipitation, topsoil organic carbon content and top soil pH) were used to simulate the potential distribution of each species.

**Figure 2:**
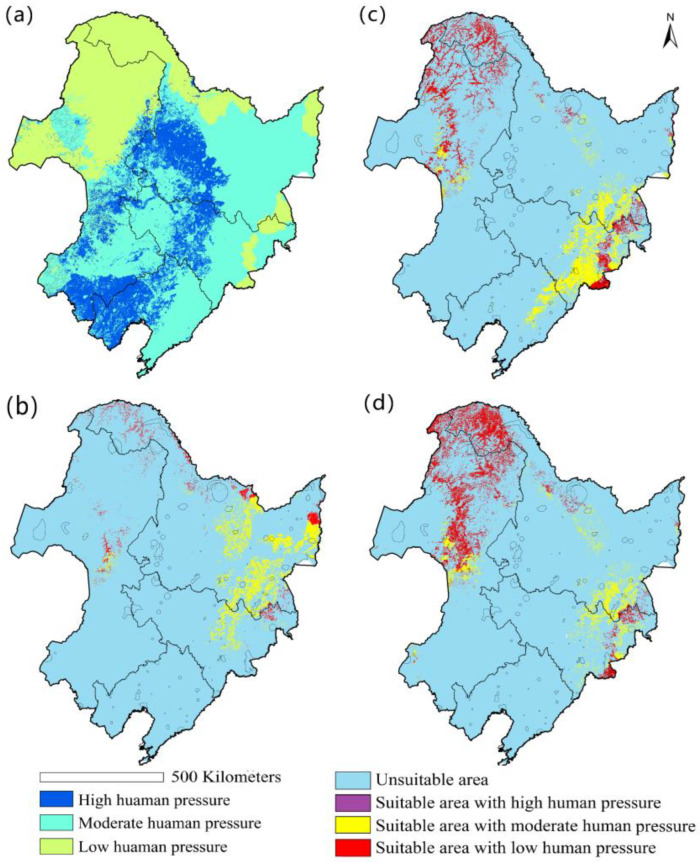
(**a**) Map of the human activities level in northeast China; (**b**) habitat suitability for *C. calceolus*; (**c**) habitat suitability for *C. macranthum*; (**d**) habitat suitability for *C. guttatum*.

Human excavation and habitat destruction were considered as the main contributors of human activities that have led to the decline of *Cypripedium* [66,67,68]. In our investigation, we also found traces of plants being eaten by cattle and sheep after grazing (Table S1). Therefore, we used population density, and the presence of pastures and farms, as the main indicators of human pressure. Human pressure values range from 0 to 19 (Table 1), and these values were divided into three categories denoting the level of impact: low (value ≤ 4), moderate (4 < value ≤ 10) and high (value > 10) impact (Figure 2a). The vegetation of northeast China was used to classify vegetation types into vegetations suited to support *Cypripedium* populations (coniferous forest, deciduous forest, mixed forest, meadow and swamp) and unsuitable vegetation types (Figure S1b). Because the investigated *Cypripedium* species only grow in a few specific vegetation types (Table S1) [55], the local vegetation conditions were taken as additional ecological factors limiting the distribution of the three species. Finally, the potential distribution area of *Cypripedium* was the merged data set of the predicted distribution by the Maxent prediction result and the area of suitable vegetation for *Cypripedium*.

#### 2.3.2. Model Evaluation

We used the receiver operating characteristic (ROC) analysis with the area under the ROC curve (AUC) index to evaluate model performance [69,70]. The ROC curve is a graph consisting of two axes; the x-axis represents the false positive fraction and is called 1-specificity, and the y-axis shows the true positive fraction named sensitivity [69]. AUC values usually vary between 0.5 and 1.0. An AUC value ≥ 0.8 indicates that the model can obtain good prediction results.

#### 2.3.3. Threshold Selection

There are many thresholds that can be used to transform the continuous probability data into binary data (presence/absence), including a value of 0.8 [71,72], the minimum predicted value [58], the 10th percentile training presence threshold [73] or the maximum value of the sum of sensitivity and specificity (MSS) [74]. Here, we used MSS as the threshold to transform the continuous suitability data into binary data. The probability distribution was reclassified into unsuitable and suitable area by judging grid values smaller or greater than MSS. For each run, 20% of the data were used as test points. Maxent currently has six feature classes: linear, product, quadratic, hinge, threshold and categorical features, and we chose the linear, product and quadratic features according to our sample size [64]. We chose the best “regularization multiplier” based on model performance (AUC). The maximum number of background points was set to 10,000 and replicated 20 times.

## 3. Results

### 3.1. Model Performance and Key Environmental Variables

The mean AUC in this study, including the test data and training data of the Maxent model with nine environmental variables, ranged from 0.810 to 0.873 (Table S2), which means that the model gave good predictions. The distribution of the three *Cypripedium* species in northeast China was strongly associated with topography (elevation and slope) and climate (isothermality, temperature seasonality, maximum temperature of warmest month, precipitation of driest quarter and seasonality of precipitation). In terms of topography, elevation had a larger influence on the distribution of *C. macranthum*, while slope had a larger influence on the distribution of *C. calceolus* and *C. guttatum*. The distribution of the three orchid species was most strongly affected by the maximum temperature of the warmest month, with relative contributions between 18.1% and 21% (Figure S4). The distribution of *C. guttatum* was also strongly affected by topsoil organic carbon content and top soil pH, while the distribution of the other two orchids was less affected by soil factors. The highest probability of occurrence was found at sites with a maximum temperature of the warmest month of 2.56 °C, an elevation higher than 500 m and slope larger than 6° (Figure S6).

### 3.2. Suitable Habitats of Cypripedium in Northeast China

**Figure 3:**
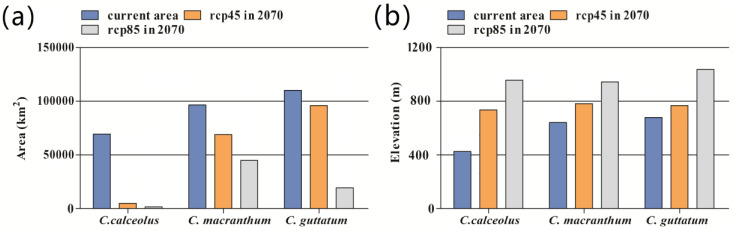
(**a**) The current and predicted area of suitable habitats under different climate change scenarios for three *Cypripedium* species in 2070 in northeast China. (**b**) The current and predicted mean elevation of suitable habitats for three *Cypripedium* species in northeast China under different climate change scenarios.

The estimated area of suitable habitat of the three species of *Cypripedium* in northeast China varied between 69,436 and 110,158 km^2^ (Figure 3a). Suitable habitats of *C. calceolus* were mainly distributed in the central and northern parts of the Changbai Mountains, the southeastern part of Xiao Xing’an Ling and few areas in Da Xing’an Ling. Most of the potential distribution was located in the Heilongjiang province and a few areas in Liaoning (Figure 2b). The suitable habitats of *C. macranthum* were mainly distributed in the central and southern parts of the Changbai Mountains, followed by sporadic occurrences in the northern and central part of Da Xing’an Ling and a few areas in Xiao Xing’an Ling (Figure 2c). The suitable habitats of *C. guttatum* occurred mainly in the central and northern parts of Da Xing’an Ling, and there were a few suitable habitats in the Changbai Mountains and Xiao Xing’an Ling (Figure 2d).

### 3.3. The Impact of Human Pressure on the Distribution of Cypripedium

Human activities were most pronounced in the northeast Plain, with no suitable area for *Cypripedium* remaining. Moderate human activities were observed in the central part of the Changbai Mountains (Figure 2). Because most of the suitable habitats of *C. calceolus* were located in this area, more than 75% of its potential habitats was subject to moderate human pressure (Figure 2b and Figure S5). Suitable habitats of *C. macranthum* (Figure 2c) and *C. guttatum* (Figure 2d) mainly occurred in Da Xing’an Ling and Xiao Xing’an Ling, where human activities were less abundant.

### 3.4. The Suitable Area of Cypripedium within Nature Reserve Area

Total reserve area in the study area comprised about 82,308 km^2^, accounting for less than 7% of the total study area. Within the reserve area, little suitable habitat was observed: 1986 km^2^ for *C. calceolus* (mainly in the Liangshui, Qixinglazi and Yueyahu nature reserve), 4417 km^2^ for *C. macranthum* (mainly in the Longwan, Jingyu and Changbai Mountains nature reserve) and 3276 km^2^ for *C. guttatum* (mainly in the Changbai Mountains, Huzhong and Nanwenghe nature reserve). Most of the protected areas are coniferous forests, broad-leaved forests or mixed coniferous and broad-leaved forests.

### 3.5. Future Suitable Habitats Prediction of Cypripedium

**Figure 4:**
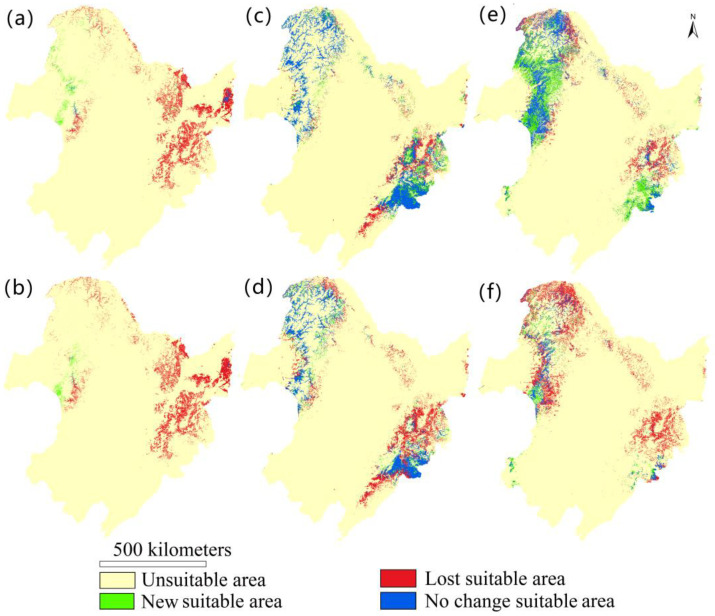
Predicted changes in habitat suitability for the three *Cypripedium* species under two climate change scenarios (rcp4.5 and rcp8.5) by 2070. Changes in habitat suitability for *Cypripedium calceolus* under (**a**) rcp4.5 and (**b**) rcp8.5; for *Cypripedium macranthum* under (**c**) rcp4.5 and (**d**) rcp8.5, and *Cypripedium guttatum* under (**e**) rcp4.5 and (**f**) rcp8.5. Rcp4.5 is a scenario that stabilizes radiative forcing at 4.5 W m^−2^ in the year 2100 without ever exceeding that value by employment of a range of technologies and strategies for reducing greenhouse gas emissions. The rcp8.5 scenario corresponds to a nominal anthropogenic forcing of 8.5 W m^−2^ by 2100, with emissions of CO_2_ following an exponential growth trajectory throughout the 21st century.

By 2070, the extent of suitable habitats for the three *Cypripedium* species will significantly decrease in the study area (Figure 3a and Figure 4). Under both climate change scenarios, most of the suitable habitats of *C. calceolus* will disappear in the Changbai Mountains and Da Xingan Mountains and only a small fraction of suitable area will remain in the northern Changbai Mountains. However, some fragmented new suitable areas will appear on the western Da Xing’an Ling. For *C. macranthum*, most of the habitats in Da Xing’an Ling will remain under the two climatic scenarios, but some suitable areas in the Changbai Mountains will disappear, especially under rcp8.5. Under rec4.5, some suitable areas of *C. guttatum* are predicted to disappear in the northern part of the Changbai Mountains, while new suitable areas would appear in Da Xing’an Ling. Most suitable areas of *C. guttatum* will disappear under rcp8.5. Compared with the current distribution, suitable habitats of the three species of *Cypripedium* will move to higher elevations under both climate scenarios. This was most evident for *C. calceolus*, with an increase in elevation from 420 m to 743 m (rcp4.5) and 950 m (rcp8.5) (Figure 3b).

## 4. Discussion

### 4.1. Ecological Niche of Cypripedium

Our field surveys demonstrated that the three studied *Cypripedium* species mostly occur in cool and humid forests with a gentle terrain and high altitude. The analyses showed that forests with substantial light penetration through the forest canopy, a sparse shrub and herb layer and a deep soil humus characterized most of the sites where we encountered *Cypripedium* populations in northeast China. Model simulations showed that these habitats were mainly found in mountainous areas. These results are in accordance with the results of Wan et al. [75], who also showed that *C. calceolus* was mainly distributed in the northern part of the Changbai Mountains. However, our results showed that suitable habitats of *C. calceolus* were found at many other places in the Changbai Mountains and also occurred widely in Da Xing’an Ling and Xiao Xing’an Ling, suggesting that the species has a much wider potential distribution than pervious analyses have indicated. Notwithstanding, 48 distribution records were used in Wan’s research; their predictions had limitations due to sampling collection bias, which may have led to inaccurate predictions of areas of suitable habitat [76]. Increased awareness of the implications of spatial bias in surveys will therefore substantially improve predictions of species distributions [76].

Although the three *Cypripedium* species have a similar life history, their distribution areas did not completely overlap, suggesting they pose somewhat different requirements towards the environmental conditions that allow long-term persistence and survival. *Cypripedium macranthum* was mainly distributed in the south-central part of the Changbai Mountains, while *C. calceolus* was mainly found in the north-central part of the Changbai Mountains and *C. guttatum* in Da Xing’an Ling. Maximum temperature of the warmest month (Bio5) appeared to have the largest impact on the distribution of the three *Cypripedium* species, indicating that climatic conditions have a determining influence on their distribution. A previous study has shown that high summer temperatures may increase the costs of respiration in *Cypripedium* [77]. In addition, precipitation of the driest quarter (Bio 17) appeared to be also important for the distribution of *Cypripedium*, suggesting that spring snow cover can have a protective effect on plant growth and provides sufficient moisture for growth [77]. Besides climatic conditions, we also found that soil conditions had an impact on the distribution of the investigated lady’s slipper orchids. Whether this is driven by a direct impact on the growth of the orchids or by altering mycorrhizal availability [75] warrants further research.

### 4.2. Future Protection under Climate Change and Human Pressure

Our simulations further showed that under the predicted changes in climatic conditions the suitable areas of the three species will move to higher elevations. Particularly, under rcp8.5, suitable areas will disappear at lower elevations. However, it remains unclear whether the species will be able to disperse to higher elevation sites without active human intervention (e.g., seed introduction). At the same time, the increased fragmentation and disappearance of the original habitats caused by climate change may have a negative impact on the future prospects of extant populations of *Cypripedium* within the study area. Considering the long time before *Cypripedium* plants start to reproduce by seeds [68,78], once habitats become unsuitable, it may take a long time before viable populations successfully establish in new suitable habitats. However, pronounced variation in microclimatic conditions in environments with complex terrains could mitigate the impact of climate change in the short term [79], because plant species may temporarily escape from regional climate change by short-distance migration to local micro-refugia [80]. Given that *Cypripedium* populations were mainly confined to mountainous areas, short-distance migration could to some extent slow down the negative impact of climate change on *Cypripedium*. Nonetheless, the pronounced loss of suitable habitat due to climate change may pose a serious threat to the long-term survival of populations of *Cypripedium* in the study area.

Human pressure appeared to have a smaller impact on the distribution of the studied *Cypripedium* species. Only in the Changbai Mountains was a substantial amount of suitable habitat of *C. calceolus* affected by moderate human pressure, while for *C. macranthum* and *C. guttatum* suitable habitats in Da Xing’an Ling and Xiao Xing’an Lingn were less affected by human pressure. These results indicate that human activities will most likely have a greater impact on the future survival of *C. calceolus* than on that of the other two species. Therefore, future conservation planning and actions should particularly pay attention to protection of suitable habitats of *C. calceolus*. Given that at present only a very small fraction of the potential distribution area of *C. calceolus* is located within existing nature reserves, protecting additional areas is a key priority to reassure the long-term viability of these species [75]. For the conservation of *Cypripedium*, the Da Xing’an Ling area appears to be the best region to set up new conservation areas since it was suitable to support the three species. Extending existing protected areas with novel suitable areas can be an ideal starting point for continued conservation in situ. Selecting sites with a slightly higher elevation than the current nature reserves can also contribute to the long-term survival of *Cypripedium* and mitigate predicted long-term climate changes.

Apart from human pressure and climatic conditions, other biotic and abiotic factors may affect the distribution of plant species, including vegetation [80], presence of pollinators [42] or suitable mycorrhizal fungi [41,81]. For example, favorable vegetation for the establishment of seedlings of *C. macranthum* includes narrow-leaved, medium-sized grasses, sedges, herbs, mosses and prostrate mat-forming shrubs. In general, the presence of these plants provides suitable moisture, temperature and light conditions at the soil surface for *C. macranthum* [80]. However, detailed data on vegetation or pollinator communities were unavailable for the entire region. Similarly, even though next-generation genomic methods have the potential to provide information about the suite of mycorrhizal fungi in the soil to support orchid populations and to drive niche differentiation in orchids, large-scale assessments of the distribution of mycorrhizal fungi are still largely lacking [82]. Future studies are therefore needed to unravel the role of mycorrhizal fungi in determining the large-scale distribution of these plants.

## 5. Conclusions

Maxent and ArcGIS were used to understand the effects of human pressure and climate change on the distribution of three *Cypripedium* species in northeast China. Our results showed that the maximum temperature of the warmest month, altitude, slope and seasonality of precipitation had important effects on the distribution of the investigated *Cypripedium* species. Human pressure had a significant impact on the distribution of *C. calceolus*, but negligible effects on the distribution of *C. macranthum* and *C. guttatum*. Predicted changes in climate will drive *Cypripedium* populations to higher elevation sites, although the complex microclimate of mountains may mitigate the negative effects of climate change. Based on these results, future conservation programs should focus on selecting reserve sites at higher altitudes and investigating whether assisted migration is needed for seeds to establish at these sites.

## Figures and Tables

**Table 1:** The environmental variables used to predict the potential geographic distribution of *Cypripedium*.

Variables	Classification	Description	Data Source	Details
Bio1	Climate	Annual Mean Temperature	Worldclim database	
Bio2	Climate	Mean Diurnal Range	Worldclim database	
Bio3	Climate	Isothermality	Worldclim database	
Bio4	Climate	Temperature Seasonality	Worldclim database	
Bio5	Climate	Maximum Temperature of Warmest Month	Worldclim database	
Bio6	Climate	Minimum Temperature of Coldest Month	Worldclim database	
Bio7	Climate	Temperature Annual Range	Worldclim database	
Bio8	Climate	Mean Temperature of Wettest Quarter	Worldclim database	
Bio9	Climate	Mean Temperature of Driest Quarter	Worldclim database	
Bio10	Climate	Mean Temperature of Warmest Quarter	Worldclim database	
Bio11	Climate	Mean Temperature of Coldest Quarter	Worldclim database	
Bio12	Climate	Annual Precipitation	Worldclim database	
Bio13	Climate	Precipitation of Wettest Month	Worldclim database	
Bio14	Climate	Precipitation of Driest Month	Worldclim database	
Bio15	Climate	Precipitation Seasonality	Worldclim database	
Bio16	Climate	Precipitation of Wettest Quarter	Worldclim database	
Bio17	Climate	Precipitation of Driest Quarter	Worldclim database	
Bio18	Climate	Precipitation of Warmest Quarter	Worldclim database	
Bio19	Climate	Precipitation of Coldest Quarter	Worldclim database	
ABT	Climate	Annual Biotemperature	Worldclim database	
CI	Climate	Coldness Index	Worldclim database	
WI	Climate	Warmth Index	Worldclim database	
|WI-CI|	Climate	Absolute Value of Warmth Index - Coldness Index	Worldclim database	
Ele	Topography	Elevation	Geospatial Data Cloud	
Slo	Topography	Slope	Geospatial Data Cloud	
Asp	Topography	Aspect	Geospatial Data Cloud	
OC	Soil	Topsoil Organic Carbon Content	Harmonized World Soil Database	The percentage of organic carbon in topsoil
pH	Soil	Topsoil pH	Harmonized World Soil Database	Measured in a soil–water solution, it is a measure for the acidity and alkalinity of the soil
Veg	Vegetation	Vegetation Type	Data Center for Resources and Environmental Sciences	Distribution of 11 vegetation types in China
Built	Human activity	Built Environments	Human Footprint maps	All areas mapped as built given a score of 10
Pop	Human activity	Population Density	Human Footprint maps	Pressure score = 3.333 × log (population density + 1)
Nig	Human activity	Night-time Density	Human Footprint maps	Equal quintile bins
Crop	Human activity	Croplands	Human Footprint maps	All areas mapped as crops given a score of 7
Pas	Human activity	Pasture	Human Footprint maps	All areas mapped as pasture given a score of 4
Roa	Human activity	Roads	Human Footprint maps	500 m either side of roads given a direct pressure score of 8 Starting 500 m out from a road, a pressure score of 4 exponentially decaying out to 15 km
Rail	Human activity	Railways	Human Footprint maps	500 m either side of railways given a direct pressure score of 8 Starting 500 m out from a road, a
Nav	Human activity	Navigable Waterways	Human Footprint maps	pressure score of 4 exponentially decaying out to 15 km

## Data Availability

The data presented in this study are available on request from the corresponding author.

## Supplementary Materials

The following are available online at https://www.mdpi.com/2223-7747/10/1/84/s1. Table S1: The location of field survey and the characteristic of *Cypripedium* in northeast China. Table S2: The AUC of Maxent for *Cypripedium* in northeast China. Figure S1: Nature reserves in northeast China and Suitable vegetation area and unsuitable vegetation area for *Cypripedium*. Figure S2: PCA analysis of 19 bioclimates of *Cypripedium*. Figure S3: Habitats of *Cypripedium* in northeast China. Figure S4: Estimates of relative contributions of the nine environmental variables. Figure S5: The suitable habitat area under different human pressure level and in reserve area for *Cypripedium*. Figure S6: The response curve of *Cypripedium* in Maxent.
