# Supplementary material for: The Impact of Human Pressure and Climate Change on the Habitat Availability and Protection of Cypripedium (Orchidaceae) in Northeast China
Source: Plants (Basel). 2021 Jan 2;10(1):84. doi: 10.3390/plants10010084 (PMC7824597; doi:10.3390/plants10010084)
Supplement: Supplementary file 1 [file plants-10-00084-s001.pdf]

Supplementary materials

Table S1

The location of field survey and the characteristic of *Cypripedium* in northeast China.

Number of Sample	Species	Location	Elevation (m)	Slope (°)	Vegetation Type	Top Soil pH	Grazing traces
1	*C. calceolus,C. macranthum, C. guttatum*	Mudanjiang City, Heilongjiang Province	455	5	Boradleaf Forest	5.7	No
2	*C. calceolus, C. macranthum, C. guttatum*	Mudanjiang City, Heilongjiang Province	547	5	Mixed forest	5.6	Yes
3	*C. calceolus, C. macranthum, C. calceolus C. guttatum*	Mudanjiang City, Heilongjiang Province	469	8	Boradleaf Forest	5.7	Yes
4	*C. macranthum*	Benxi City, Liaoning Province	523	21	Boradleaf Forest	5.7	No
5	*C. macranthum*	Fushun City, Liaoning Province	738	10	Boradleaf Forest	5.9	No
6	*C. calceolus, C. macranthum,*	Yichun City, Heilongjiang Province	345	8	Needleleaf Forest	6.2	No
7	*C. macranthum, C. guttatum*	Yichun City, Heilongjiang Province	370	7	Boradleaf Forest	5.6	No
8	*C. macranthum*	Yanbian City, Jilin Province	412	12	Needleleaf Forest	7.1	No
9	*C. calceolus, C. macranthum, C. guttatum*	Yanbian City, Jilin Province	682	12	Boradleaf Forest	5.5	No
10	*C. guttatum*	Yanbian City, Jilin Province	1975	18	Meadow	4.9	No
11	*C. macranthum*	Yanbian City, Jilin Province	1426	3	Meadow	5.2	No
12	*C. guttatum*	Hulun Buir City, Inner Mongolia Province	482	5	Boradleaf Forest	5.8	No
13	*C. guttatum*	Da Xing'an montains, Heilongjiang Province	478	6	Mixed forest	5.2	No

14	*C. guttatum*	Da Xing'an montains, Heilongjiang Province	642	7	Mixed forest	5.3	No
15	*C. guttatum*	Da Xing'an montains, Heilongjiang Province	394	10	Needleleaf Forest	5.8	No
16	*C. macranthum*	Da Xing'an montains, Heilongjiang Province	404	12	Needleleaf Forest	5.5	No
17	*C. calceolus, C. macranthum, C. guttatum*	Da Xing'an montains, Heilongjiang Province	342	14	Needleleaf Forest	6.5	No
18	*C. guttatum*	Hulun Buir City, Inner Mongolia Province	645	5	Needleleaf Forest	5.4	No
19	*C. macranthum*	Hulun Buir City, Inner Mongolia Province	742	4	Swamp	6.2	No
20	*C. macranthum, C. guttatum*	Hing'an League, Inner Mongolia Province	1077	9	Boradleaf Forest	6.2	No

Table S2

The AUC of Maxent for *Cypripedium* in northeast China.

Species	AUC	rcp45 in 2070s	rcp85 in 2070s
C. calceolus	0.871	0.839	0.833
C. macranthum	0.868	0.810	0.816
C. guttatum	0.873	0.876	0.839

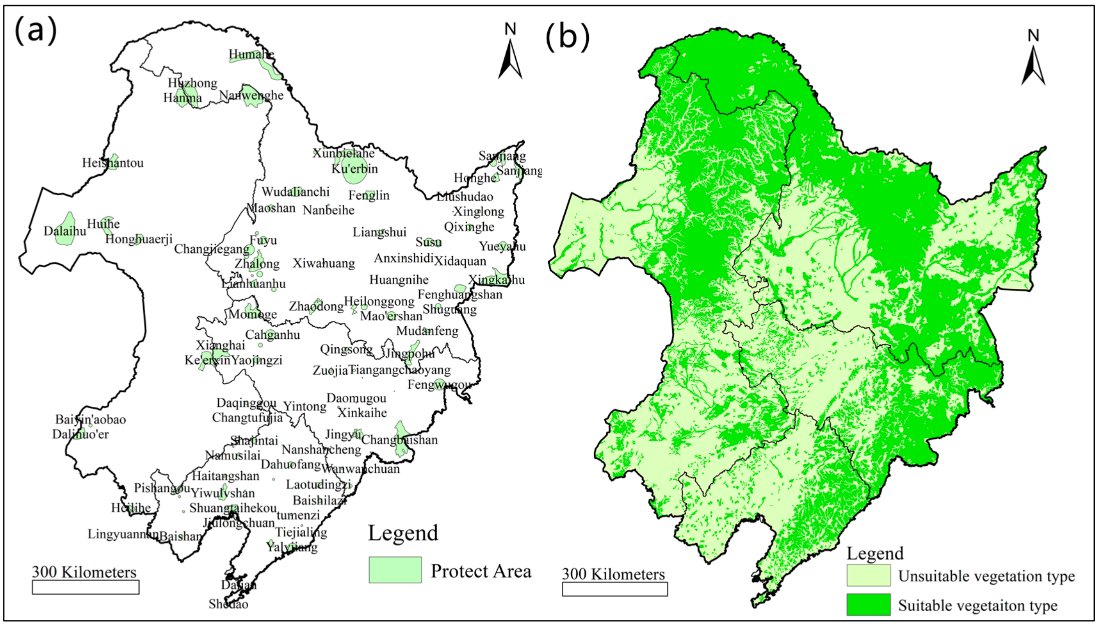

Figure A1. (a) Nature reserves in northeast China, (b) Suitable vegetation area and unsuitable vegetation area for *Cypripedium*.

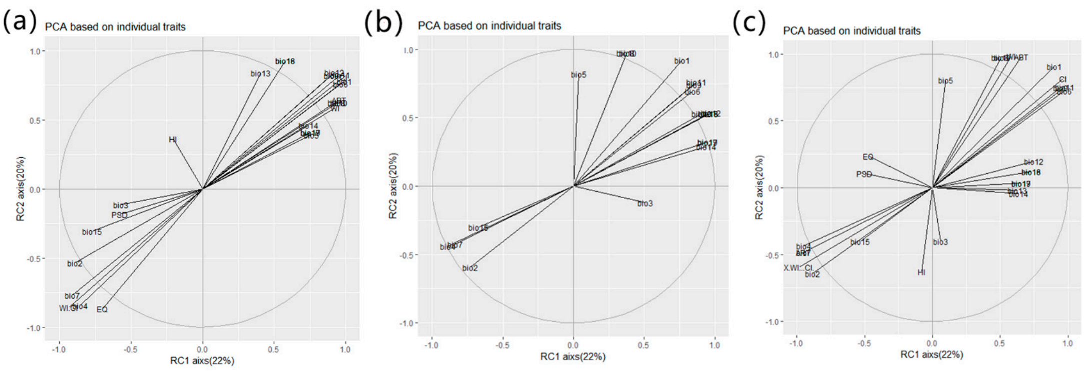

Figure S2. (a) PCA analysis of 19 bioclimates of *C. calceolus*, (b) PCA analysis of 19 bioclimates of *C. macranthum*, (c) PCA analysis of 19 bioclimates of *C. guttatum*.

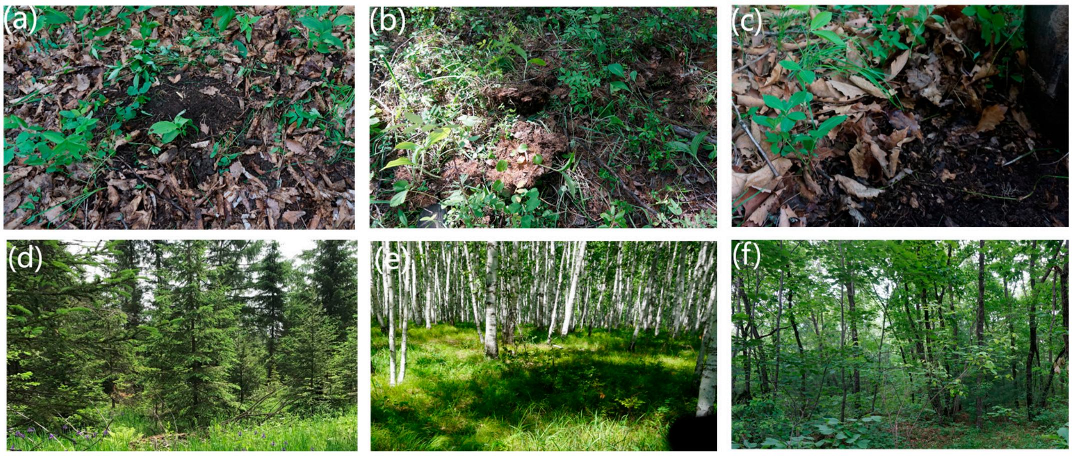

Figure S3. (a) (b) (c)Soil and humus layer of *Cypripedium* in northeast China, (d) (e) (f) Vegetation of *Cypripedium* in northeast China.

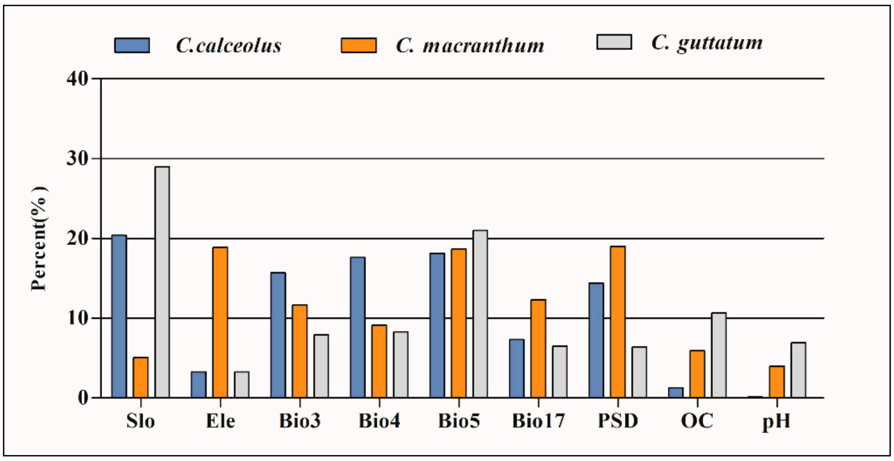

Figure S4. Estimates of relative contributions of the nine environmental variables Slope (Slo), Elevation (Ele), Isothermality (Bio3), Temperature Seasonality (Bio4), Maximum Temperature of Warmest Month (Bio5), Precipitation of Driest Quarter (Bio17), Seasonality of Precipitation (PSD) top soil organic carbon content (OC) and top soil pH (pH) by Maxent.

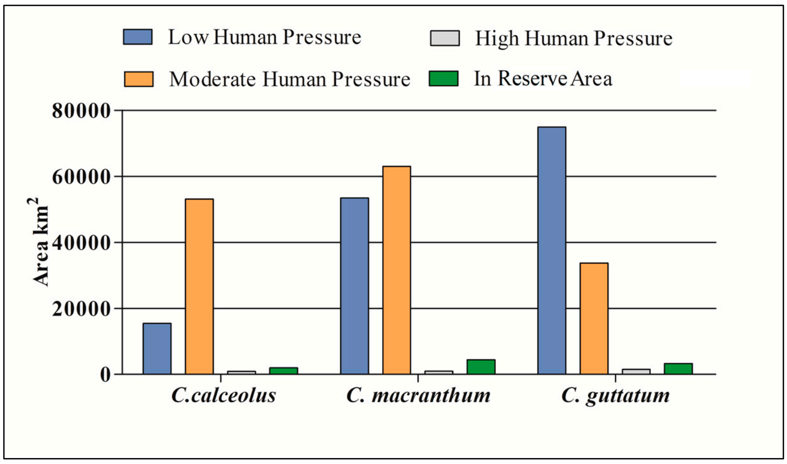

Figure S5. The suitable habitat area under different human pressure level and in reserve area for *Cypripedium*.

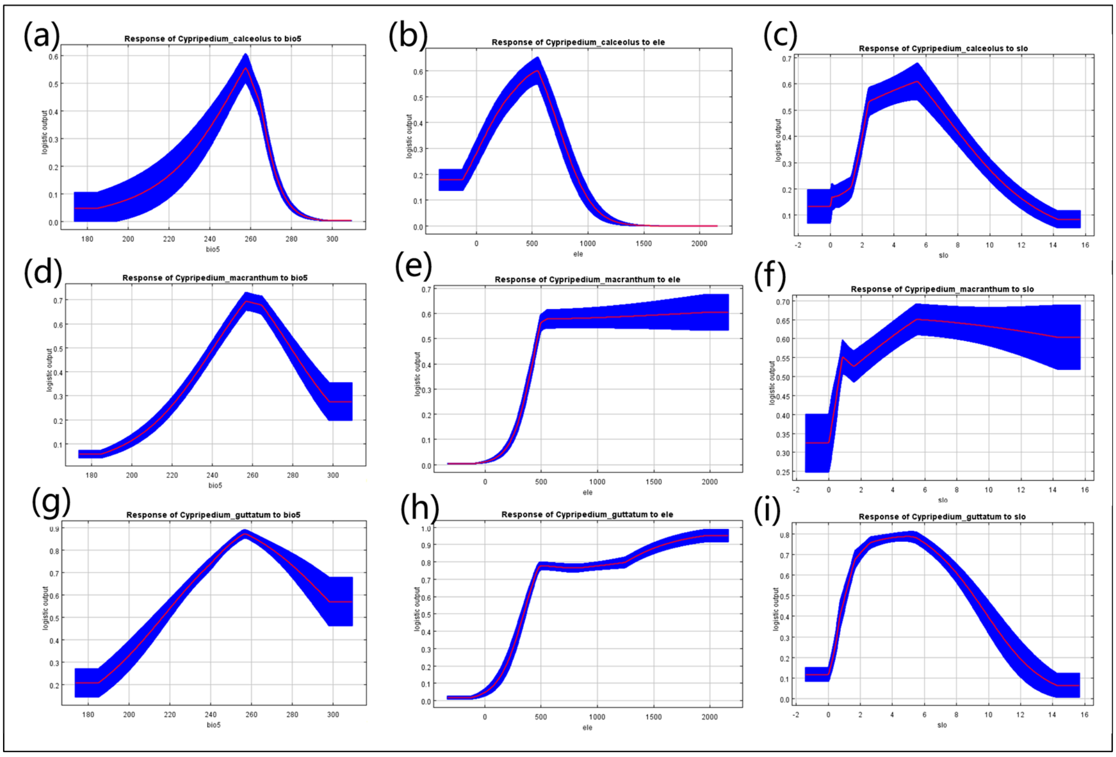

Figure S6. (a) The response curve of *C. calceolus* to Maximum Temperature of Warmest Month (Bio5), (b) The response curve of *C. calceolus* to Elevation (Ele), (c) The response curve of *C. calceolus* to Slope (Slo), (d) The response curve of *C. macranthum* to Maximum Temperature of Warmest Month (Bio5), (f) The response curve of *C. macranthum* to Elevation (Ele), (e) The response curve of *C. macranthum* to Slope (Slo), (g) The response curve of *C. guttatum* to Maximum Temperature of Warmest Month (Bio5), (h) The response curve of *C. guttatum* to Elevation (Ele), (i) The response curve of *C. guttatum* to Slope (Slo).
